# Sonochemical Functionalization of SiO_2_ Nanoparticles with Citric Acid and Monoethanolamine and Its Remarkable Effect on Antibacterial Activity

**DOI:** 10.3390/ma18020439

**Published:** 2025-01-18

**Authors:** Iván Toledo-Manuel, Marissa Pérez-Alvarez, Gregorio Cadenas-Pliego, Christian Javier Cabello-Alvarado, Guadalupe Tellez-Barrios, Carlos Alberto Ávila-Orta, Antonio Serguei Ledezma-Pérez, Marlene Andrade-Guel, Pascual Bartolo-Pérez

**Affiliations:** 1 Centro de Investigación en Química Aplicada (CIQA), Blvd. Ing. Enrique Reyna H. No. 140, Saltillo 25294, Coahuila, Mexico; ivan.toledo.d22@ciqa.edu.mx (I.T.-M.); guadalupe.tellez.d24@ciqa.edu.mx (G.T.-B.); carlos.avila@ciqa.edu.mx (C.A.Á.-O.); antonio.ledezma@ciqa.edu.mx (A.S.L.-P.); marlene.andrade@ciqa.edu.mx (M.A.-G.); 2 CONAHCyT-Centro Investigación en Química Aplicada (CIQA), Blvd. Enrique Reyna 140, Saltillo 25294, Coahuila, Mexico; christian.cabello@ciqa.edu.mx; 3 Centro de Investigación y de Estudios Avanzados del IPN-Unidad Mérida, Departamento de Física Aplicada, Mérida 97310, Yucatán, Mexico; pascual.bartolo@cinvestav.mx

**Keywords:** sonochemical, SiO_2_ nanoparticles, antimicrobial activity

## Abstract

Nanoparticles (NPs) are excellent antibacterial agents due to their ability to interact with microorganisms at the cellular level. However, their antimicrobial capacity can be limited by their tendency to agglomerate. Functionalizing NPs with suitable ligands improves their stability and dispersion in different media and enhances their antibacterial activity. The present work studied the functionalization of SiO_2_ NPs using the sonochemical method and the Influence of organic ligands on antimicrobial activity (AA). The organic ligands studied were citric acid (CA) and monoethanolamine (MEA). X-ray diffraction (XRD) and X-ray photoelectron spectroscopy (XPS) results confirmed the amorphous structure of SiO_2_ NPs and their functionalization. Thermogravimetric analysis (TGA) and X-ray photoelectron spectroscopy (XPS) showed that functionalization with MEA (SiO_2_-MEA NPs) is more favored compared to AC (SiO_2_-CA NPs), and the organic ligand content was 34.42% and 28.0%, respectively. Fourier-transform infrared spectroscopy (FTIR) and RAMAN spectroscopy results confirmed the functionalization of NPs through the presence of carboxyl and amino groups. Scanning electron microscopy (SEM), transmission electron microscopy (TEM), and zeta potential results showed that functionalization of SiO_2_ NPs helped to improve their dispersion and prevent their agglomeration. Furthermore, the results of antibacterial activity against *Staphylococcus aureus* and *Escherichia coli* showed that the functionalization provided a significant improvement in the antibacterial activity (AA) of the SiO_2_ NPs, where the SiO_2_-CA NPs showed the highest activity, with a 99.99% inhibition percentage at concentrations of 200 ppm against both *E. coli* and *S. aureus* strains. The AA is maintained at high concentrations of 1200 ppm, which is essential in applications requiring high percentages of biocidal NPs, such as marine coatings.

## 1. Introduction

In the constant search for solutions to address public health challenges related to the proliferation of pathogenic microorganisms and increasing antibiotic resistance, nanoparticles (NPs) have proven to be promising antibacterial agents. Among them, silicon dioxide nanoparticles (SiO_2_ NPs) have attracted considerable attention due to their properties, including chemical stability, low toxicity, mechanical strength, and antimicrobial capacity. These characteristics have enabled their use in various applications, from electronics and drug delivery to anti-corrosion and antibacterial coatings [1,2]. Although some studies on SiO_2_ NPs suggest excellent antimicrobial activity (AA), others present contradictory results. Moreover, most of these studies are limited and do not determine the minimum inhibitory concentration (MIC) [3,4]. In general, SiO_2_ NPs exhibit lower AA with respect to other NPs, such as Ag, Cu, and TiO_2_, among others [4,5]. To our knowledge, the antimicrobial activity of SiO_2_ NPs, either alone or functionalized, has not been studied in detail. In most studies, SiO_2_ NPs are used in combination with other nanomaterials, acting mainly as a support for NPs considered to have high AA (Ag, Cu, TiO_2_, ZnO, etc.), to avoid their agglomeration and maintain their AA efficiency [4,5,6,7].

In particular, the antimicrobial capacity of SiO_2_ NPs is enhanced by the presence of silanol groups (Si-OH) located on their surface. These groups give the NPs a hydrophilic nature, improving their interaction with bacteria. This interaction facilitates antimicrobial action by promoting better adhesion and penetration into the bacterial surface, which interferes with their vital functions and inhibits their growth [8]. However, the effectiveness of SiO_2_ NPs in antimicrobial applications may be compromised due to their tendency to agglomerate, a phenomenon resulting from their high surface energy. The agglomeration phenomenon can modify the surface area, size, and shape of the NPs, reducing their ability to penetrate the bacterial membrane and damage the genetic material [9,10]. Functionalization of NPs is presented as an effective solution to overcome these limitations, allowing not only the prevention of agglomeration but also the adjustment of their properties for specific applications [11,12,13,14]. By reducing the agglomeration of NPs, their incorporation into different media is facilitated. For example, using functionalized NPs in polymers improves their dispersion and increases the properties of interest [15,16,17,18]. Functionalization of SiO_2_ NPs has been reported to prevent agglomeration and improve their antimicrobial properties. For example, Wen et al. [19] demonstrated that SiO_2_ NPs functionalized with a block copolymer of poly(2,2,2-trifluoroethylmethacrylate) or PTFEMA and poly(sulfobutylmethacrylate) or PSBMA offered superior antifouling performance compared to non-functionalized NPs. Likewise, Dong et al. [20] reported that the functionalization of SiO_2_ NPs with N-halamine significantly improved their antibacterial performance against Gram-positive and Gram-negative bacteria.

One of the technologies used to functionalize NPs is sonication or ultrasonic irradiation. During this process, bubbles are generated that grow and collapse violently. Although cavitation collapse is short-lived and the energy released by each bubble is minimal, its cumulative effect can lead to extremely high levels of energy being released into the liquid, causing an increase in temperature and pressure in the medium [14,21]. This cavitation activity presents several advantages in the functionalization of NPs. First, it promotes the activation of chemical bonds, facilitating the incorporation of functional groups on the surface of NPs. In addition, it contributes to improving the dispersion of NPs, which enables better contact between reagents [22,23,24]. Another advantage of this technique is its low environmental impact since it reduces reaction times and eliminates the need to use hazardous reagents [25]. Ultrasonic irradiation can be carried out by direct contact with the sample or indirectly through the walls of the container containing the sample. In the direct contact method, an ultrasonic probe that incorporates a piezoelectric transducer in the form of a tip is used and placed directly in contact with the object or surface to be treated. The probe emits ultrasonic waves directly to the point of application, that is, to the interface between the material and the molecular modification zone. This allows greater precision and control over the intensity and direction of the ultrasound compared to the indirect method, in which the piezoelectric transducer is located on the wall of the tank. The generated ultrasonic waves are transmitted through the liquid penetrating the walls of the container containing the sample. This indirect method’s main limitation is its low power transmission to the medium [26,27].

Various organic ligands can be used to functionalize NPs. Among them, citric acid (CA) and monoethanolamine (MEA) are suitable ligands. CA, a weak acid widely found in nature [28], is considered a non-toxic compound used in different applications, such as a chelating agent, antioxidant, flavoring agent, and antibacterial agent [28,29]. CA comprises three carboxyl functional groups linked through ester bonds or by electrostatic interactions with the hydroxyl groups of NPs [28,30,31]. In addition, these carboxyl groups provide suitable binding sites for the adhesion of additional molecules, allowing a more specific functionalization according to the desired application [30,31,32]. In previous studies, CA has been used as a ligand for the functionalization of NPs, improving their magnetic properties, preventing their agglomeration, increasing their biocompatibility, and improving their antibacterial properties [30,33,34]. However, using environmentally friendly technologies, the specific strategy of functionalizing SiO_2_ NPs with CA has been less explored. On the other hand, MEA is a primary amine containing a hydroxyl group (OH) and an amino group (-NH_2_) capable of forming covalent bonds or electrostatic interactions with NPs [35,36,37]. MEA is commonly used as a stabilizing agent in synthesizing nanomaterials due to its ability to prevent their agglomeration [38,39,40]. Furthermore, it has been shown that the functionalization of NPs with amines increases their antimicrobial capacity. The amino group in the functionalized NPs facilitates the rupture of the bacterial cell wall, allowing NPs to enter the cell. Once inside, NPs damage the genetic material of the bacteria, which ultimately causes its death [37].

In the present study, the effect of the type of organic ligand on the functionalization of SiO_2_ NPs was investigated using an environmentally friendly method, ultrasonic irradiation. An ultrasound probe with an output power of 750 W and a frequency of 20 kHz was used to explore how CA and MEA affect the stability, dispersion, and degree of functionalization of the NPs. Furthermore, the effect of each ligand on the antibacterial activity of SiO_2_ NPs against *Escherichia coli* (*E. coli*) and *Staphylococcus aureus* (*S. aureus*) strains was evaluated.

## 2. Materials and Methods

### 2.1. Materials

SiO_2_ NPs from Sigma-Aldrich (St. Louis, MO, USA) with a particle size <20 nm and a purity of 99.5% were used. Citric acid (99.5%) (C_6_H_8_O_7_) in powder form and monoethanolamine (99%) (C_2_H_7_NO), both from Jalmek Scientific (Nuevo Leon, Mexico), were used as organic ligands. Distilled water with a pH between 6.5 and 7 was used to prepare the solutions. In the evaluation of antimicrobial activity, the microorganisms used were *Escherichia coli* ATCC-25922 and *Staphylococcus aureus* ATCC-29213, were provided by American Type Culture Collection (ATCC), (Manassas, VA, USA).

### 2.2. Functionalization of SiO_2_ NPs

The functionalization of SiO_2_ NPs was carried out similarly to what was reported by [14]. The procedure is described below: 2.0 g of SiO_2_ NPs were weighed into a beaker, and 100 mL of a CA solution with a concentration of 0.5 M was added. Then, the sample was homogenized on a grill at 20 rpm, maintaining a temperature of 80 °C for 20 min. Subsequently, the obtained mixture was subjected to ultrasound irradiation using a Cole-Palmer sonicator (Vernon Hills, IL, USA) with an output power of 750 W, a wave amplitude of 50%, and a frequency of 20 kHz for 60 min at room temperature. After this, the sample was heated at 80 °C with continuous stirring for 20 min at 20 rpm. Finally, the resulting solution was centrifuged at 12,000 rpm for 20 min. The solid obtained was washed with abundant distilled water until a pH between 6.5 and 7.0 was reached and then dried in a vacuum oven at 100 °C for 24 h. The procedure for functionalizing SiO_2_ NPs with MEA was similar to that performed with CA; in this case, 100 mL of a MEA solution at a concentration of 1.7 M was used. Table 1 presents the identification of the samples.

### 2.3. Characterization

X-ray diffraction (XRD) patterns were obtained using a Bruker D8 Advance eco diffractometer (Bruker, Billerica, MA, USA), with a scanning range of 20 to 80° (2θ) and a scanning speed of 0.02% per second. K-alpha copper radiation with a wavelength of 1.54 Å and a D-teX Ultra detector were used, operating at an intensity of 35 kV and a voltage of 25 mA.

A thermogravimetric analyzer (TGA), model Q500 (TA instruments., New Castle, PA, USA), was used to evaluate the thermal stability of the NPs. The operating conditions were a heating rate of 10 °C/min in a nitrogen atmosphere with a 50 mL/min gas flow in the temperature range of 25 to 600 °C. After 600 °C, an oxygen atmosphere was used to achieve complete combustion of the organic material.

Differential scanning calorimetry (DSC) analyses were performed using a DuPont Instruments 951 calorimeter (TA instruments, New Castle, PA, USA) at a heating rate of 10 °C/min over a temperature range of 25 to 110 °C for the first heating and 25 to 300 °C for the second heating.

Attenuated total reflectance Fourier-transform infrared (ATR-FTIR) spectroscopy analyses were performed using a Magna Nicolet i550 (Termo Fisher Scientific., Waltham, MA, USA) in the 400–4000 cm^−1^ range.

Raman spectroscopy analyses were performed on a Horiba Xplora micro-confocal instrument (Horiba., Minami-ku, Kyoto, JPN). Experiments were performed at an excitation wavelength of 785 nm. The laser was focused through a 50X objective with a numerical aperture of 0.75.

To analyze the surface composition and oxidation states of non-functionalized (NF-NPs) and functionalized (F-NPs) SiO₂ NPs with the different organic ligands, the X-ray photoelectron spectroscopy (XPS) technique was used. The analyses were performed in a Thermo Scientific spectrophotometer (Thermo Scientific, Waltham, MA, USA), model K-ALPHA, with a monochromatic X-ray source, a binding energy of 0–1350 eV, and a depth of 400 μm. No treatment was performed on the samples prior to analysis.

Scanning electron microscopy (SEM) was used to obtain high-resolution images of the surface of the NPs, allowing the observation of the morphology and size distribution of the NPs. The analyses were carried out using Phillips XL30 ESEM equipment (Philips, Portland, OR, USA). with an acceleration voltage of 5–25 keV. No pretreatment of the samples was performed prior to the analysis. The transmission electron microscopy (TEM) technique was used to investigate the structure of the NPs, for which a high-resolution FEI-Titan 80–300 KV electron microscope was used (FEI Company, Hillsboro, OR, USA).

Zeta potential analyses were performed using Microtrac Zeta Check equipment (Microtrac Inc., Montgomeryville, PA, USA). The samples were analyzed at a concentration of 2.5 M, using distilled water as a dispersion medium. Prior to the analysis, the samples were dispersed using Qsonica brand Q700CA ultrasonic equipment (Qsonica, LLC, CT, USA) with an amplitude of 40% for 5 min.

### 2.4. Antimicrobial Activity

Antibacterial activity assays were performed using the JIS Z 2801, antimicrobial products test for antimicrobial activity and efficacy standards [41]. The strains of bacteria studied were *Escherichia coli (E. coli)* Gram (-) and *Staphylococcus aureus (S. aureus)* Gram (+). The analysis was carried out using the plate-pouring diffusion method and trypticase soy agar. First, the bacteria were inoculated in trypticase soy agar until a concentration of 100,000 colony-forming units per milliliter (CFU/mL) was obtained, assigned as the concentration at time zero. Subsequently, F-NP and NF-NP solutions were prepared in duplicate at concentrations of 1400, 1100, 800, 500, and 200 mg/L. Then, 200 µL of the bacteria suspension (100,000 CFU/mL) was added to each of these concentrations and incubated for 24 h at 37 °C.

Serial dissolutions were then made from each of the sample concentrations. To do this, 1 mL of the 1400 mg/L solution was taken and placed in a tube containing 9 mL of sterile saline solution. This tube was homogenized for 5 min on a vortex mixer and labeled as tube 10⁻^1^. This procedure was repeated by transferring 1 mL from tube 10⁻^1^ to another tube with 9 mL of sterile saline solution, homogenizing it for 5 min, and labeling it as tube 10⁻^2^. This process was continued until a 10⁻⁵ dissolution was achieved. For each dissolution tube, a 1 mL sample was taken and placed in a Petri dish, to which 25 mL of trypticase soy agar was added. These plates were incubated for 24 h at 37 °C. The antibacterial activity (R) was calculated using Equation (1) [41], and the percentage of inhibition was determined using Equation (2) [17,42].

(1) $$R=\left[log\left(\frac{B}{A}\right)-log\left(\frac{C}{A}\right)\right]$$

(2) $$Inhibition\;(\%)=\frac{A-C}{C}\;\times \;100$$

where *R* is the antibacterial activity value, *A* is the average CFU/mL at time zero, *B* is the average CFU/mL formed on the control plate (bacteria solution without NPs) after 24 h of incubation, and *C* is the average CFU/mL of the NPs-F and NPs-NF after 24 h of incubation.

## 3. Results and Discussion

### 3.1. X-Ray Diffraction (XRD)

**Figure 1:**
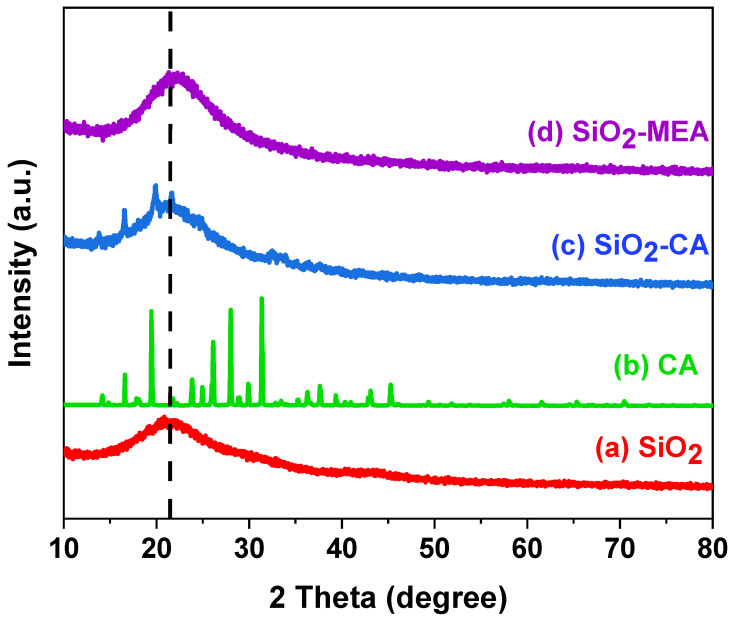
XRD patterns of (a) pure SiO_2_ NPs, (b) CA, (c) SiO_2_-CA NPs, and (d) SiO_2_-MEA NPs.

Figure 1 shows the XRD patterns of the F-NPs and NF-NPs with the different ligands. The XRD pattern of SiO_2_ NPs exhibited an amorphous peak centered at 2θ = 21.1° [10]. In contrast, the XRD pattern of SiO_2_-CA NPs presented three crystalline peaks at 2θ = 16.59°, 19.97°, and 21.72°, which are attributed to the characteristic peaks of the CA crystal structure [43,44]. These diffraction patterns for CA agree with the data reported by the International Center for Diffraction Data (JCPDS, No. 00-022-1536) [45].

On the other hand, the diffractograms obtained from SiO_2_ NPs and SiO_2_-MEA NPs showed minor differences. The diffractogram of the sample of SiO_2_-MEA NPs showed a slightly thinner amorphous peak and shifted towards higher 2θ angles. This evidence suggests the interaction of SiO_2_ NPs and MEA. Although MEA does not present X-ray diffraction peaks, its presence is detected through the changes observed in the amorphous peak of SiO_2_ NPs.

### 3.2. Thermogravimetric Analysis (TGA)

**Figure 2:**
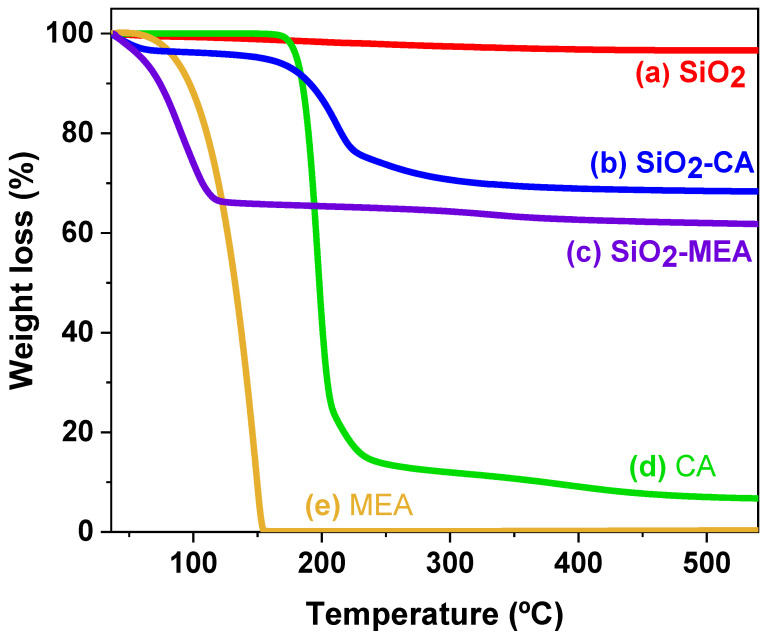
Thermograms of (a) SiO_2_ NPs, (b) SiO_2_-CA NPs, (c) SiO_2_-MEA NPs, (d) CA, and (e) MEA.

Figure 2 shows the thermograms of the F-NPs and NF-NPs (SiO_2_) and the ligands CA and MEA. The NF-NPs present an initial weight loss of 0.66% in the range of 37 °C to 100 °C, corresponding to the loss of volatile compounds and moisture [2]. In addition, a second weight loss of 2.66% is observed in the range of 176 °C to 550 °C, attributable to the dehydroxylation of the OH groups on the surface of the NPs [46,47,48].

The thermogram of the CA sample showed two main instances of weight loss. The first loss of 78.12% was at the maximum degradation temperature (T_max_) of 192.49 °C, which is attributed to the decomposition of the functional groups of the CA. The second loss of 15.21% is recorded near 214.2 °C and is related to the thermal decomposition of the by-products formed during the first degradation of the CA. Similar data have been reported [43,49,50]. On the other hand, the thermogram of the MEA shows a single weight loss of 99.87% at a T_max_ of 136.95 °C, which is consistent with its reported degradation temperature [51,52].

The weight loss data of F-NPs and NF-NPs at different temperatures are presented in Table 2. It is observed that functionalization with MEA was found to be more effective compared to CA, with a ligand content of 34.42% and 28.0%, respectively.

When comparing the T_max_ of the F-NPs with the different ligands, it was determined that the SiO_2_-CA NPs (T_max_ of 200 °C) present higher thermal stability compared to the SiO_2_-MEA NPs (T_max_ of 93.60 °C), with mass losses of 31.71% and 38.24%, respectively, at 600 °C. These results suggest that the functionalization of SiO_2_ NPs with CA produces a nanocomposite with higher thermal stability than MEA, which is explained by the tendency of MEA to form quaternary ammonium compounds, which present low thermal stability [36].

### 3.3. Differential Scanning Calorimetry (DSC)

**Figure 3:**
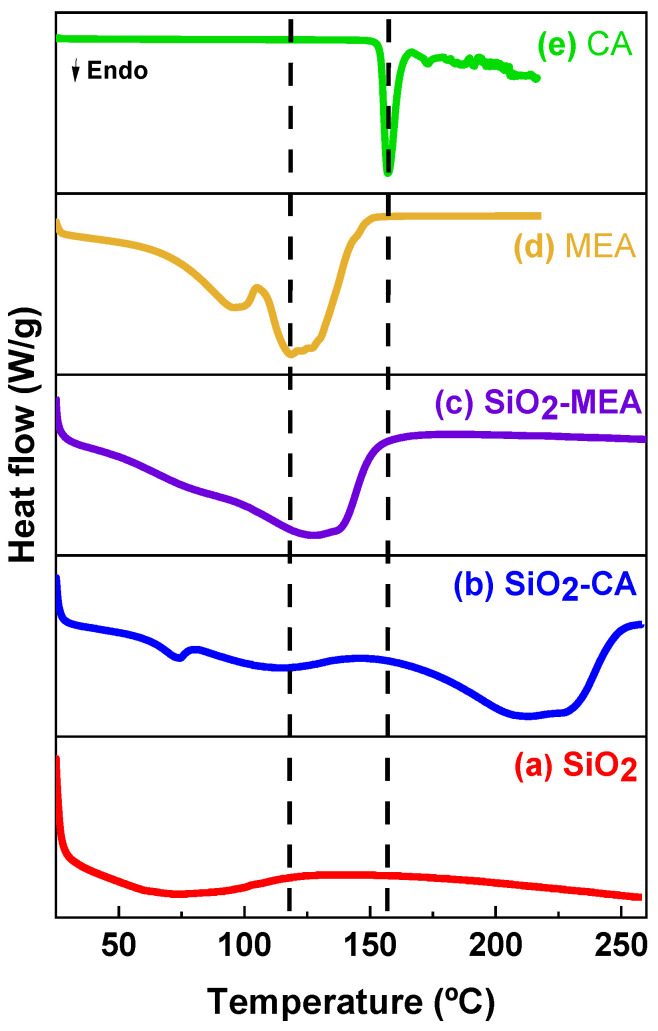
DSC thermograms of (a) SiO_2_ NPs, (b) SiO_2_-CA NPs, (c) SiO_2_-MEA NPs, (d) MEA, and (e) CA.

DSC thermograms of the F-NPs, NF-NPs, and organic ligands are shown in Figure 3. The SiO_2_ NPs show a broad endothermic transition attributed to water loss and volatile compounds [46,48]. On the other hand, MEA shows two endothermic peaks at 96 °C and 122 °C, corresponding to the loss of water and decomposition of the MEA, respectively. After this transition, a deviation from the baseline to approximately 153 °C occurred, suggesting a degradation process of the MEA ligand [51]. The DSC thermogram of the CA ligand presented a single narrow endothermic peak at 157 °C, which is consistent with the melting temperature reported in the literature [43,53]. After melting, the deviation from the baseline was also observed up to approximately 215 °C, suggesting the thermal degradation of the CA ligand.

The DSC thermogram of SiO_2_-CA NPs shows the stability observed in the TGA analysis (Figure 2). SiO_2_-CA NPs showed three thermal transitions. The first was observed at around 73 °C and is attributable to the presence of volatile compounds. The second endothermic transition at around 120 °C corresponds to water molecules adsorbed on the surface of SiO_2_ NPs and possibly dehydroxylation due to the condensation of hydroxyl groups (-OH). The third, at 220 °C, is attributed to the removal of water and the degradation of CA [43,53].

The DSC thermogram of SiO_2_-MEA NPs showed a broad endothermic transition around 120 °C, attributable to the initial decomposition temperature of the MEA ligand, which was very similar to that observed in the pure MEA sample. The thermograms of F-NPs suggest that SiO_2_-CA NPs showed higher thermal stability than SiO_2_-MEA NPs. This may be because the CA molecule contains multiple carboxyl groups (-COOH) capable of forming stronger bonds with the surface of SiO_2_ NPs. The intermolecular interactions between the hydroxyl (OH) groups of SiO_2_ NPs and the carboxyl groups of the CA ligand could contribute to this higher thermal stability [28,30].

### 3.4. Attenuated Total Reflectance Fourier-Transform Infrared Spectroscopy (ATR-FTIR)

**Figure 4:**
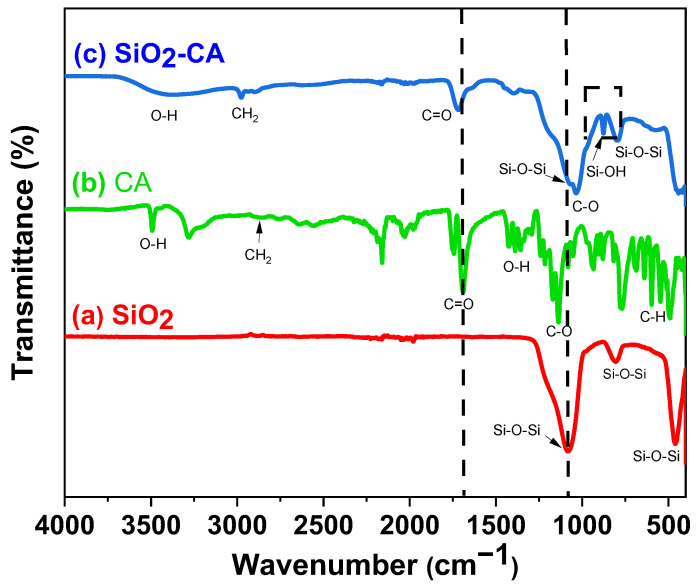
FT-IR spectra of (a) SiO_2_ NPs, (b) CA, and (c) SiO_2_-CA NPs.

Figure 4 shows the ATR-FTIR spectra of the SiO_2_ NPs, SiO_2_-CA NPs, and CA ligand samples. The ATR-FTIR spectrum of the sample of SiO_2_ NPs presented three absorption peaks at 1080 cm^−1^, 804 cm^−1^, and 467 cm^−1^, corresponding to the stretching vibration of the Si-O bond that is related to the Si-O-Si fragment [54,55].

The ATR-FTIR spectrum of the CA sample showed absorption bands in the range of 485 to 3000 cm^−1^, corresponding to the characteristic functional groups of its molecular structure. The absorption bands at 3489 cm^−1^ and 3276 cm^−1^ indicate the presence of undissociated OH groups, while the band in the region of 2870 cm^−1^ corresponds to the stretching vibrations of the methylene (CH_2_) groups [11,31]. The absorption bands at 1745 cm^−1^ and 1690 cm^−1^ are attributed to the asymmetric and symmetric stretching modes of the C=O bond in the carboxyl group (COOH); the presence of more than one band in this region confirms the formation of a solid-state dimer. The bands centered at 1400 cm^−1^ are attributed to the bending of the O-H bonds of the C-OH fragment, while the band around 1127 cm^−1^ is associated with the stretching vibrations of the C-O bond. Finally, the band at 770 cm^−1^ is attributed to the vibration of the C-H bond. Furthermore, different vibration modes of the COOH fragment are located between 485–807 cm^−1^. The ATR-FTIR spectrum of CA is generally complex due to the different intermolecular hydrogen bonds that may exist [56].

The ATR-FTIR spectrum of the sample of SiO_2_-CA NPs showed the same bands as those of the SiO_2_ NPs and CA ligand samples. The adsorption bands are broader and show slight shifts (Figure 4). In addition, two new bands were observed: a broad band located at 3400 cm^−1^ and a thinner one at 877 cm^−1^, both attributed to the vibrations of the Si-OH bond that is characteristic of the silanol (Si-OH) group [54,56,57]. The ATR-FTIR spectrum of the sample of SiO_2_-CA NPs is less complex than that presented by the CA ligand. This suggests the loss of intermolecular interactions in the CA and the formation of new interactions with the surface of SiO_2_ NPs. This observation supports the functionalization of SiO_2_ NPs and is confirmed by the shifts observed in the bands corresponding to the COOH and Si-O-Si groups [11,58,59]. The formation of silyl ester is ruled out since no shift towards lower wavelengths of the carbonyl group band was observed, and the aqueous reaction medium does not favor the condensation reaction.

**Figure 5:**
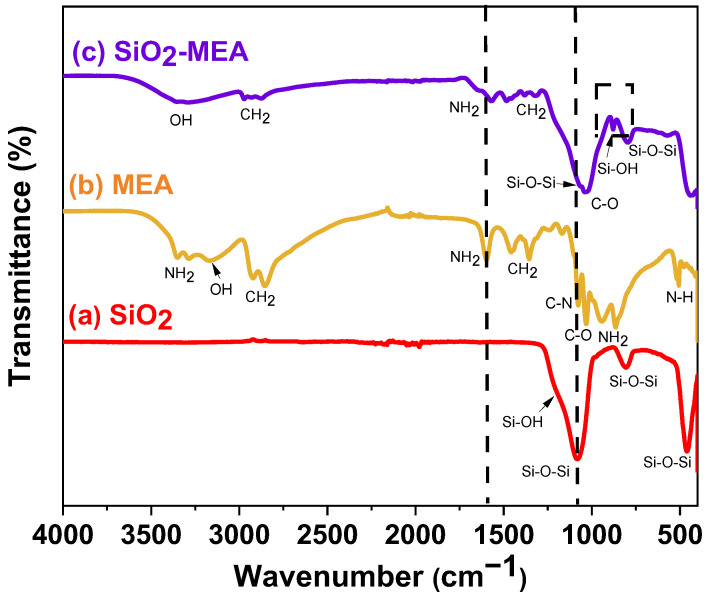
FTIR spectra of (a) SiO_2_ NPs, (b) MEA, and (c) SiO_2_-MEA NPs.

The ATR-FTIR spectra of the MEA ligand and SiO_2_-MEA NPs are shown in Figure 5. The spectrum of the MEA ligand exhibits peaks located at 3354 cm^−1^, 3278 cm^−1^, 1596 cm^−1^, 939, and 863 cm^−1^, corresponding to the asymmetric and symmetric stretching vibrations of the NH2 bond, while the bands at 2928 cm^−1^, 2848 cm^−1^, 1451 cm^−1^, and 1348 cm^−1^ are attributed to the asymmetric and symmetric stretching vibrations of the CH_2_ bond [39,60,61,62]. The stretching vibrations of the C-N and C-O bonds are evident at 1076 cm^−1^ and 1029 cm^−1^, respectively [63,64].

The ATR-FTIR spectrum of the sample of SiO_2_-MEA NPs showed the same bands observed in the SiO_2_ NPs and MEA ligand samples. The adsorption bands are broader and present slight shifts in the characteristic peaks of the NH_2_ and Si-O-Si groups (Figure 5). In addition, a new small band was observed at 1523 cm^−1^, which could indicate the presence of the ammonium group (RNH_3_^+^). The NH bond stretching band, which is generally broad and intense, is located between 2800 cm^−1^ and 3200 cm^−1^. ATR-FTIR analysis again confirmed the formation of the silanol group (Si-OH), which presented bands at 3357 cm^−1^ and 879 cm^−1^, corresponding to the vibrations of the OH and Si-OH bonds, respectively [54,56,57].

ATR-FTIR analysis of the sample of SiO_2_-MEA NPs confirmed the functionalization of SiO_2_ NPs with MEA. The slight shifts of the bands suggest that the interaction between MEA and SiO_2_ NPs is weak and possibly governed by hydrogen bonds. A similar behavior has been reported in the literature [40]. Another possible explanation is the formation of electrostatic attractions between the functional groups present, such as OH-CH_2_-CH_2_-NH_3_^+ −^(O-Si). The presence of quaternary ammonium compounds has been reported in the functionalization of metal oxides with MEA [36,65].

### 3.5. Raman Spectroscopy

**Figure 6:**
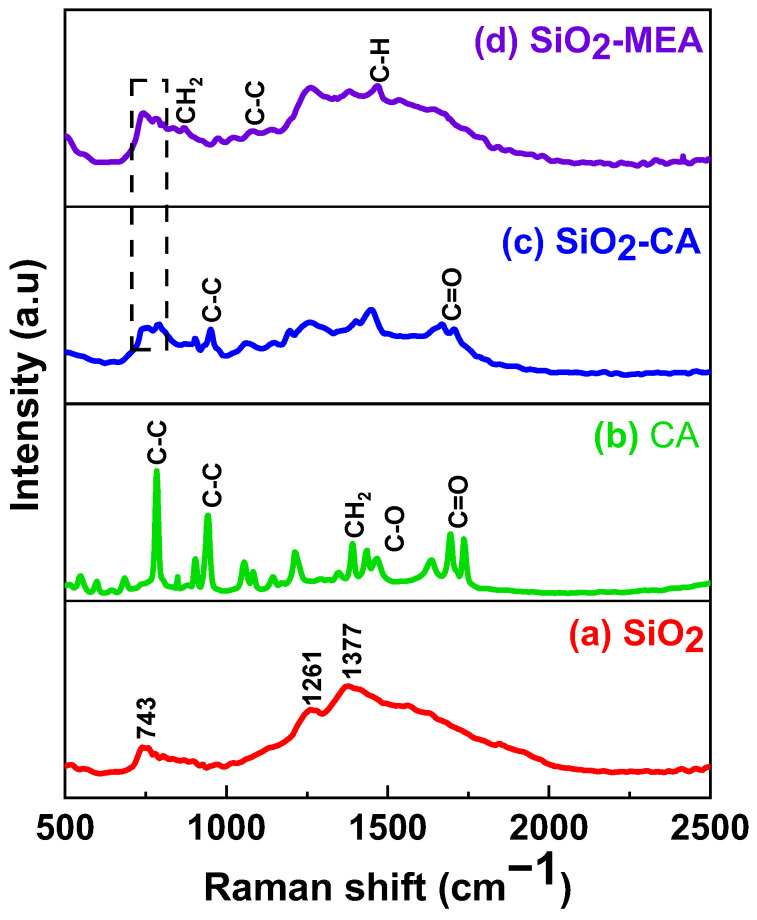
Raman spectra of (a) SiO_2_ NPs, (b) CA, (c) SiO_2_-CA NPs, and (d) SiO_2_-MEA NPs.

Figure 6a shows the Raman spectrum of the NF-NPs, where the characteristic peaks corresponding to the vibration modes of the amorphous SiO_2_ NPs are observed [66,67,68,69]. The peaks located at 743 cm^−1^ and 1261 cm^−1^ are attributed to the stretching of the Si-O-Si bond [66,67,68,70]. On the other hand, the Raman spectrum of the CA (Figure 6b) shows a peak at 949 cm^−1^ and another at 778 cm^−1^, both associated with the symmetric elongation of the C-C bond. The peak at 1387 cm^−1^ corresponds to the bending vibration of the CH_2_ group, while the peak at 1472 cm^−1^ is attributed to the presence of the C-O bond. The peak at 1690 cm^−1^ indicates the presence of a non-ionized carboxylic group, while the peak at 1742 cm^−1^ corresponds to the presence of an ionized carboxyl group [43,71,72].

The Raman spectra of the F-NPs show new and shifted peaks corresponding to the organic groups with which they are functionalized. In the Raman spectrum of the SiO_2_-CA NPs, the characteristic peaks of CA described above are observed. In addition, a marked shift of the C=O bond towards lower wavelengths is evident, going from 1690 cm^−1^ to 1669 cm^−1^ and 1738 cm^−1^ to 1709 cm^−1^, respectively. These changes suggest a possible coordination between the hydroxyl group (OH) of the SiO_2_ NPs and the carboxylate group (COO-) of the CA, as observed in the FTIR results (Figure 4). This shift has been reported in the literature [43]. On the other hand, Figure 6 (d) shows the Raman spectrum of SiO_2_-MEA NPs, where the characteristic peaks of MEA can be observed. The peak at 871 cm^−1^ is attributed to the vibration of the CH_2_ bond, the peak at 1078 cm^−1^ is attributed to the vibration of the C-C bond, and the peak at 1078 cm^−1^ is attributed to the vibration of the C-H bond [73,74,75,76]. The appearance of the peak at 786 cm⁻^1^ in the Raman spectra of F-NPs can be attributed to specific vibrations associated with ligand interactions on the NP surface. The higher intensity of this peak in SiO_2_-CA NPs, compared to SiO_2_-MEA NPs, suggests a stronger interaction between CA and SiO_2_ NPs. These interactions may include hydrogen bond formation or coordination of functional groups, such as the carboxylate groups (-COO-) of CA, with SiO_2_ NPs. These results are consistent with those obtained by FTIR (Figure 4 and Figure 5).

### 3.6. X-Ray Photoelectron Spectroscopy (XPS)

**Figure 7:**
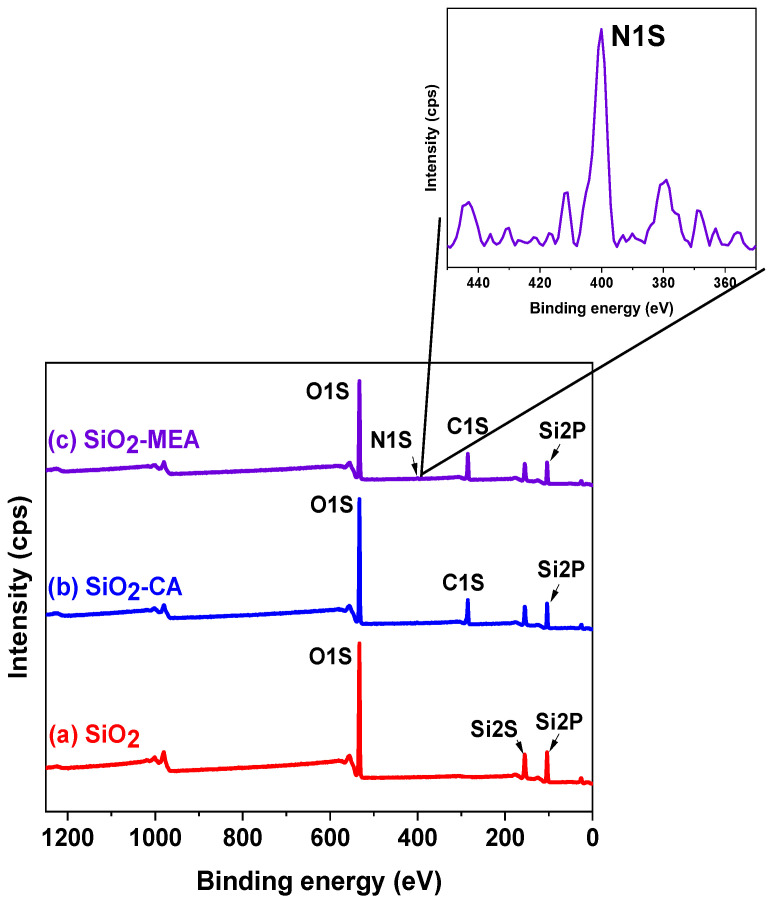
XPS spectrum of (a) SiO_2_ NPs, (b) SiO_2_-CA NPs, and (c) SiO_2_-MEA NPs.

Figure 7 shows the XPS spectra of F-NPs and NF-NPs. In the XPS spectrum of SiO_2_ NPs, three characteristic peaks are observed at 103.97 eV, 155.08 eV, and 533.17 eV, corresponding to Si2p, Si2s, and O1s, respectively. The most intense peak, with an atomic percentage of 59.53% (Table 3) assigned to O1s, is related to the Si-O bond, attributable to the OH groups present on the surface of the NPs [77,78,79,80]. SiO_2_-CA NPs presented the same characteristic peaks as SiO_2_ NPs as well as a peak around 285.26 eV, which is attributable to the C1s signal of the CA structure. On the other hand, the characteristic peaks of SiO_2_ NPs were also observed in SiO_2_-MEA NPs, with an additional peak at 400.14 eV, assigned to N1s, corresponding to the MEA structure. These values coincide with those reported in the literature [81,82,83].

Table 3 presents the atomic percentages of the F-NPs and NF-NPs obtained from XPS survey spectra. After the functionalization treatment, a decrease in the atomic percentage of the Si2p signal of the SiO_2_ NPs was observed, along with the appearance of 28.3% of C1s in the SiO_2_-CA NPs and 1.35% of N1s and 19.15% of C1s in the SiO_2_-MEA NPs. These results again confirm the functionalization of the NPs. Furthermore, when considering the carbon/silicon ratio in describing the degree of functionalization [78,84], it can be stated that the degree of functionalization was higher for the SiO_2_-MEA NPs (C/Si = 1.26) than for the SiO_2_-CA NPs (C/Si = 1.04). These results are consistent with those obtained in the TGA analyses (Table 2).

**Figure 8:**
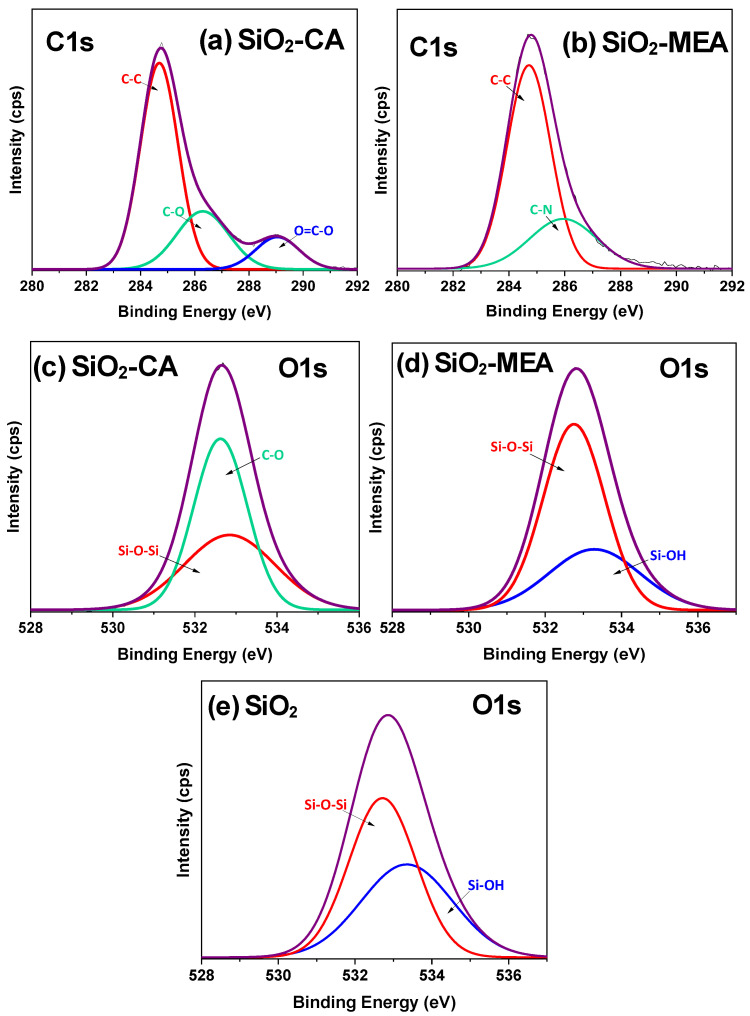
Deconvolution of (**a**) the C1s peak corresponding to SiO_2_-CA NPs, (**b**) the C1s peak corresponding to SiO_2_-MEA NPs, (**c**) the O1s peak corresponding to SiO_2_-CA NPs, (**d**) the O1s peak corresponding to SiO_2_-MEA NPs and (e) O1s peak corresponding to SiO_2_ NPs.

**Figure 9:**
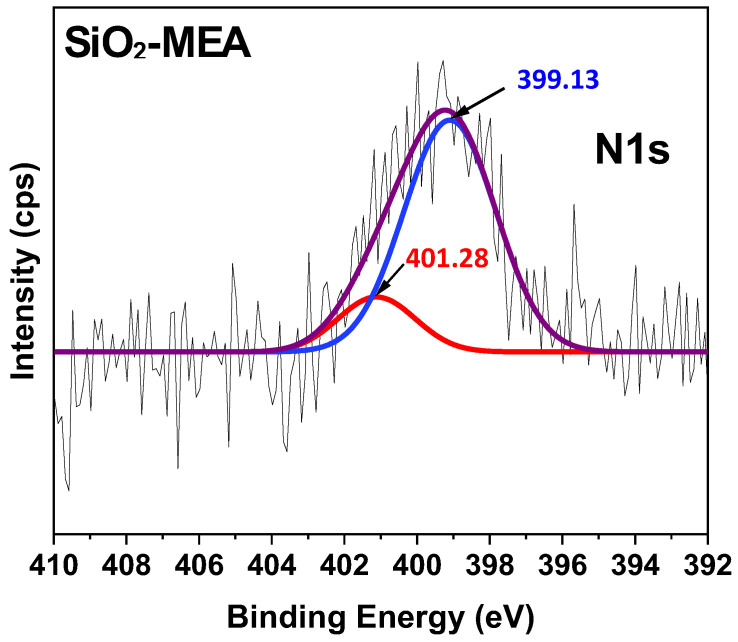
Deconvolution of the N1s peak corresponding to SiO_2_-MEA NPs.

The deconvolution of the C1s and O1s peaks of F-NPs is presented in Figure 8. For SiO_2_-CA NPs (Figure 8a,c), the peaks centered at 284.6 eV, 286.3 eV, 289.0 eV, 532.6 eV, and 532.8 eV correspond to C-C, C-O, O=C-O, C-O, and Si-O-Si bonds, respectively [79,84,85,86]. For SiO_2_-MEA NPs (Figure 8b,d), the peaks at 284.7 eV, 285.9, 532.7 eV, and 533.3 eV correspond to C-C, C-N, Si-O-Si, and Si-OH bonds, respectively [78,79,82,87,88]. In the N1s deconvolution of the sample of SiO_2_-MEA NPs (Figure 9), two signals were detected at 401.28 eV and 399.13 eV, corresponding to protonated nitrogen (-NH_3_^+^) and non-protonated nitrogen, respectively [89]. These results again confirm the functionalization of SiO_2_ NPs with CA and MEA.

The absence of the Si-OH bond in the O1s peak deconvolution of SiO_2_-CA NPs (Figure 8c), compared to SiO_2_-MEA NPs (Figure 8d), suggests a stronger interaction between SiO_2_ NPs and the CA ligand. The O1s peak deconvolution of NF NPs (SiO_2_ NPs) was performed to corroborate these results, as presented in Figure 8e. In this Figure, the presence of the Si-OH bond can be appreciated, which disappears once functionalization with the CA ligand is performed. These results indicate that the nature of the functional groups of the ligands influences the formation of new interactions with SiO_2_ NPs.

### 3.7. Scanning Electron Microscopy (SEM)

**Figure 10:**
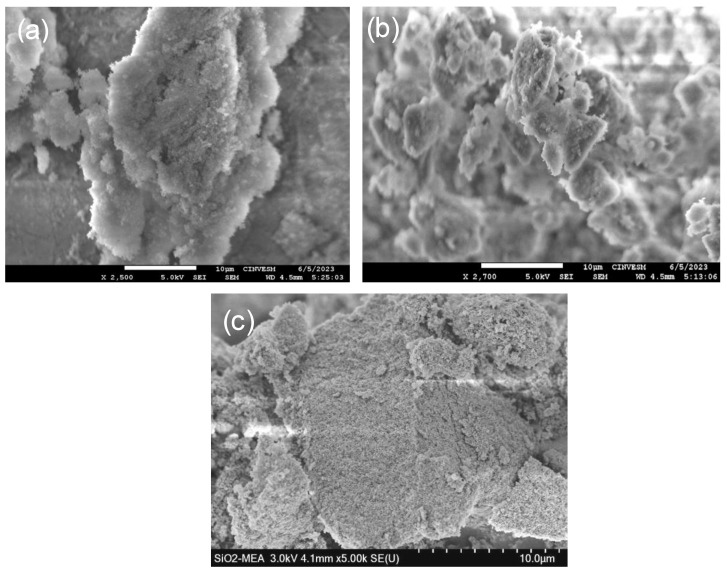
SEM images of (**a**) SiO_2_ NPs, (**b**) SiO_2_-CA NPs, and (**c**) SiO_2_-MEA NPs.

The morphological characteristics of the F-NPs and NF-NPs are presented in Figure 10. All micrographs were obtained at the same magnification of 10 µm. The images demonstrate that the SiO_2_ NPs present a non-uniform, amorphous, and slightly porous nature [54]. It is observed that the F-NPs exhibit a lower tendency to agglomerate compared to the NF-NPs, suggesting that the functionalization helped to improve their dispersion. In particular, the SiO_2_ NPs functionalized with the CA ligand show a higher dispersion and a more homogeneous distribution compared to those functionalized with the MEA ligand.

### 3.8. Transmission Electron Microscopy (TEM)

**Figure 11:**
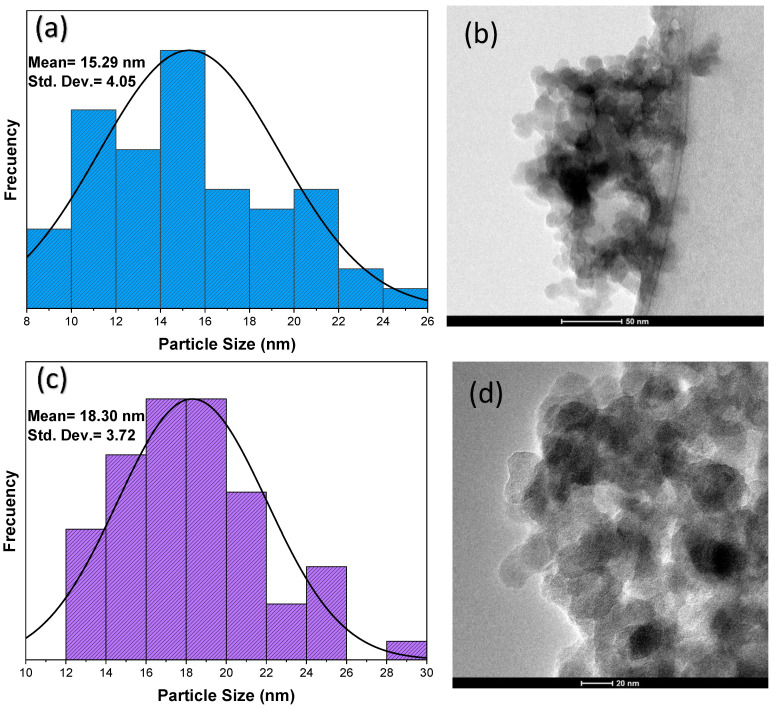
TEM images of (**a**) the histogram for SiO_2_-CA NPs, (**b**) the micrograph for SiO_2_-CA NPs, (**c**) the histogram for SiO_2_-MEA NPs, and (**d**) the micrograph for SiO_2_-MEA NPs.

Particle size histograms and TEM micrographs of the F-NPs are presented in Figure 11. In the histogram in Figure 11a, the SiO_2_-CA NPs present an average particle size of 15.29 nm. The TEM micrograph (Figure 11b) shows the spherical morphology of these NPs, which have a tendency to form small agglomerates. Individual NPs are observed to possess a well-defined shape, suggesting that functionalization with CA contributes to reducing agglomeration.

On the other hand, the histogram in Figure 11c shows that the SiO_2_-MEA NPs present an average particle size of 18.30 nm. The TEM micrograph in Figure 11c reveals the quasi-spherical morphology of these NPs, which have a higher tendency to agglomerate than the SiO_2_-CA NPs. Although individual NPs are still visible, agglomerates are more prominent, suggesting that MEA functionalization is less effective in controlling NP dispersion.

### 3.9. Zeta Potential

The surface charge of the F-NPs and NF-NPs was determined by zeta potential analysis, which is shown in Table 4. The SiO_2_ NPs present a surface charge of −70.7 mV ± 0.1, which is attributed to hydroxyl (OH) groups on their surface. On the other hand, the SiO_2_-CA NPs presented a more positive potential (−45.1 mV ± 0.2). This is possibly due to the carboxyl group forming interactions with the SiO_2_ NPs, leaving a partially positive charge on the surface.

On the other hand, SiO_2_-MEA NPs presented a much more negative zeta potential (−81.2 ± 0.2) than NF-NPs. This behavior is due to the formation of electrostatic attractions of the type OH-CH_2_-CH_2_-NH_3_^+ −^(O-Si), which increase the hydroxyl groups on the particle surface, leaving a negative surface charge. The presence of quaternary ammonium compounds was determined by ATR-FTIR (see Section 3.4) and was reported in the functionalization of metal oxides with MEA [36]. This type of attraction is weak, according to the results obtained by FTIR and Raman spectroscopy (Figure 6 and Figure 7). The effect of NP functionalization on zeta potential has been reported in the literature [90,91,92].

Zeta potential values can also provide information about the tendency of NPs to agglomerate; it has been pointed out that values below the absolute value of 30 mV can favor this phenomenon [58,93]. According to the results obtained, it can be observed that both F-NPs and NF-NPs present good dispersion stability.

### 3.10. Antimicrobial Activity

**Figure 12:**
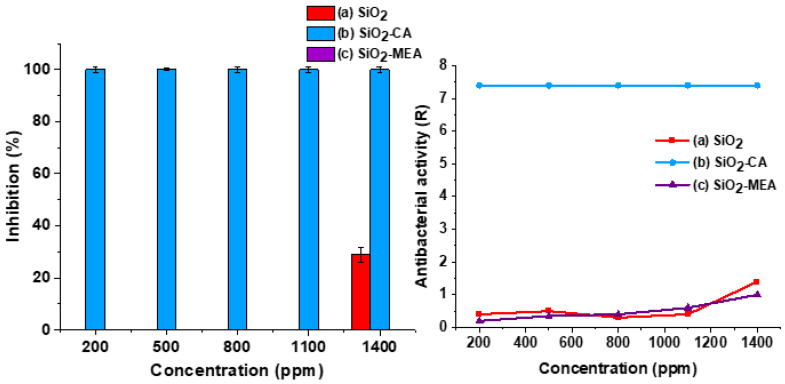
Percentage of bacterial inhibition and antibacterial activity of nanoparticles against *Escherichia coli* for (a) SiO_2_ NPs, (b) SiO_2_-CA NPs, and (c) SiO_2_-MEA NPs.

Figure 12 shows the percentages of bacterial inhibition and antibacterial activity against *E. coli* for F-NPs and NF-NPs. According to the JIS Z2801 standard [41], a product is classified as antibacterial if the antibacterial activity (R) value is greater than 2. SiO_2_ NPs (Figure 12(a)) showed low inhibition and antibacterial activity percentages, with values of 29% and 1.3, respectively, at a concentration of 1400 ppm. In contrast, SiO_2_-CA NPs (Figure 12(b)) achieved an inhibition of 99.99% and an antibacterial activity of 7.4 from concentrations of 200 ppm. On the other hand, SiO_2_-MEA NPs (Figure 12(c)) did not show inhibition percentages at any of the concentrations evaluated, with a maximum antibacterial activity of 0.9 at a concentration of 1400 ppm.

**Figure 13:**
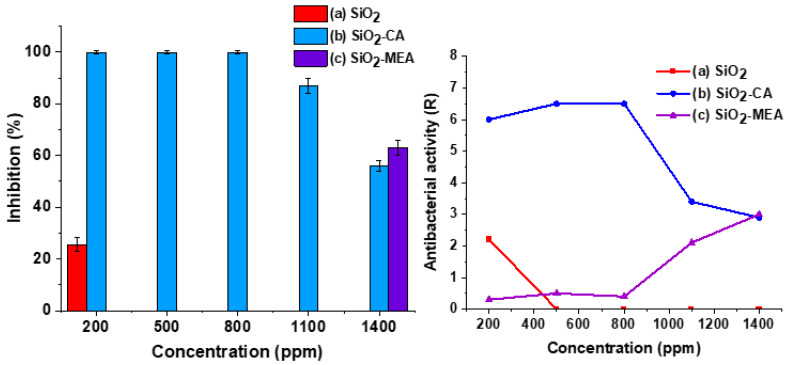
Percentage of bacterial inhibition and antibacterial activity of nanoparticles against *Staphylococcus aureus* for (a) SiO_2_ NPs, (b) SiO_2_-CA NPs, and (c) SiO_2_-MEA NPs.

Figure 13 shows the percentages of bacterial inhibition and antibacterial activity against *S. aureus* for F-NPs and NF-NPs. The non-functionalized SiO_2_ NPs showed an inhibition percentage of 25.5% and an antibacterial activity of 2.2 at a concentration of 200 ppm. In contrast, the SiO_2_-CA NPs achieved an inhibition of 99.99% and an antibacterial activity of 6.0 at the same concentration. However, as the concentration of these NPs increased, a decrease was noted in both the inhibition percentages and the antibacterial activity values.

The decrease in the inhibition percentage with increasing NP concentrations could be attributed to the agglomeration phenomenon. Agglomeration increases the size of NPs, restricting direct contact with cell walls, which is the main antibacterial action mechanism of SiO_2_ NPs. This mechanism consists of the interaction of NPs with the cell membrane, which causes its structure to rupture and destabilize, leading to the loss of cellular integrity and, eventually, to the death of the bacteria [6,94,95,96,97]. The interaction of NPs with cell walls is an interesting topic and has been reported in different studies [98,99]. Furthermore, the thicker peptidoglycan layer present in Gram-positive bacteria, compared to Gram-negative bacteria, could have acted as an additional barrier, hindering the penetration of SiO_2_-CA NPs into bacterial cells [94,100].

On the other hand, SiO_2_-MEA NPs showed a different behavior. Increasing the concentration improved their antibacterial activity, reaching an inhibition percentage of 63% at a concentration of 1400 PPM. This behavior can be explained by the electrostatic interactions between the positive charge of the cell membrane and the negative surface charge acquired by the NPs after functionalization with MEA, as observed in the zeta potential results (Table 4). These interactions favor the attraction between the NPs and the bacteria, enhancing their antibacterial action.

When comparing the results of antibacterial activity between F-NPs and NF-NPs, it was observed that NPs SiO_2_-CA achieved higher percentages of inhibition and antibacterial activity against both Gram-negative bacteria (*E. Coli*) and Gram-positive bacteria (*S. aureus*) compared to NPs SiO_2_ and NPs SiO_2_-MEA. This suggests that carboxyl groups (-COOH) effectively eliminated bacteria with negative and positive charges in their cell membranes. It is likely that the ionization of the -COOH groups favors the interaction with the cell wall, improving its antibacterial activity.

## 4. Conclusions

Functionalization of SiO_2_ NPs with CA and MEA by using the sonochemical method, a green chemistry technique, was successfully carried out. According to the TGA results, the degree of functionalization was higher for the MEA-functionalized NPs, reaching an organic ligand percentage of 34.42% compared to 28.0% for the CA-functionalized NPs.

The nature of the functional groups of the ligands significantly influenced the formation of bonds with SiO_2_ NPs, as observed in FTIR, RAMAN, and XPS analyses. When comparing the T_max_ of the F-NPs with the different ligands, it was determined that the SiO_2_-CA NPs (T_max_ of 200 °C) present a higher thermal stability compared to the SiO_2_-MEA NPs (T_max_ of 93.60 °C). This is explained by the tendency of MEA to form quaternary ammonium compounds, which present low thermal stability.

Functionalization decreased the agglomeration of SiO_2_ NPs, as evidenced by SEM and TEM results. The results obtained on antibacterial activity showed that SiO_2_-CA NPs exhibited higher antibacterial activity than SiO_2_-MEA NPs. The antibacterial activity may be related to the antibacterial properties of CA and its interaction with SiO_2_ NPs.

## Figures and Tables

**Table 1:** Sample identification.

Sample	Organic Ligand	Identification
**SiO_2_**	None	SiO_2_
**SiO_2_**	Citric acid	SiO_2_-CA
**SiO_2_**	Monoethanolamine	SiO_2_-MEA

**Table 2:** TGA Data for F-NPs and NF-NPs.

NPS	Volatile ^a^ (%)	Organic Ligand ^b^ (%)	Tmax. (°C)	Residue ^c^ (%)
**SiO_2_**	3.32	0.00	ND	96.68
**SiO_2_-CA**	3.71	28.00	200.60	68.29
**SiO_2_-MEA**	3.82	34.42	93.60	61.76
**CA**	ND	93.33	192.49	6.67
**MEA**	ND	99.87	136.95	0.14

^a^ Temperature of 35–90 °C, ^b^ Temperature of 90–600 °C, ^c^ T ≥ 600 °C, ND = not detected.

**Table 3:** Atomic percentage determined by XPS survey spectra for F-NPs and NF-NPs.

Sample	Si2p Peak (eV)	O1s Peak (eV)	C1s Peak (eV)	N1s Peak (eV)	SI2p Atomic (%)	O1s Atomic (%)	C1s Atomic (%)	N1s Atomic (%)
**SiO_2_**	103.97	533.17	ND	ND	40.47	59.53	ND	ND
**SiO_2_-CA**	103.86	533.03	285.26	ND	27.25	44.45	28.3	ND
**SiO_2_-MEA**	103.87	533.06	285.25	400.14	15.21	64.29	19.15	1.35

ND = not detected.

**Table 4:** Zeta potential data for F-NPs and NF-NPs.

Sample	Zeta Potential (mV)
**SiO_2_**	−70.7 ± 0.1
**SiO_2_-CA**	−45.1 ± 0.1
**SiO_2_-MEA**	−81.2 ± 0.2

## Data Availability

Date are contained within the article.
